# Single stimulus color can modulate vection

**DOI:** 10.3389/fpsyg.2015.00406

**Published:** 2015-04-10

**Authors:** Yasuhiro Seya, Megumi Yamaguchi, Hiroyuki Shinoda

**Affiliations:** ^1^Department of Computer and Human Intelligence, College of Information Science and Engineering, Ritsumeikan UniversityKusatsu, Japan; ^2^Graduate School of Information Science and Engineering, Ritsumeikan UniversityKusatsu, Japan

**Keywords:** vection, color, optical flow, depth perception, self-motion

## Abstract

In the present study, we investigated the effects of single color on forward and backward vection. The approaching or receding optical flow observed during forward or backward locomotion was simulated by using random dots with changing size, velocity, and disparity. The dots were presented on a black (Experiments 1 and 2) or white background (Experiment 3) in equiluminant colors; namely, white (or gray), red, yellow, green, or blue. The participant's task was to press and hold one of three buttons whenever they felt vection. The three buttons corresponded to the subjective strength of vection: strong, same, and weak relative to vection induced by the standard modulus. In Experiments 1 and 2, the participants were also asked to rate the strength and direction of vection after each trial. In Experiment 3, they rated the visibility and the perceived velocity of dot motion. Experiment 1 showed that the induced vection was stronger for the chromatic than for the achromatic dots. Particularly at low velocity conditions (±10 km/h), the vection induced for red dots was stronger than that for the other colored dots. Experiment 2 showed that the order effects of stimulus presentation could not explain the findings of Experiment 1. Experiment 3's pattern of results was similar to that of Experiment 1, and this suggested that a luminance artifact between color conditions could not account for Experiment 1's findings. These results suggest that a stimulus color can modulate vection even when a single color is added to the optical flow.

## Introduction

When a visual stimulus occupies a large part of the observer's visual field and it moves uniformly, observers often perceive body movements in the opposite direction of the stimulus motion, irrespective of whether they are actually moving or not. This phenomenon is called vection (Brandt et al., 1973). A familiar example is that, when an observer in a stationary train at a station views an adjacent moving train, the observer feels as if his/her train is moving. Self-motion perception is created by vestibular information from vestibular organs, immediately after an observer moves. However, vestibular organs respond only to the acceleration of body movements and, therefore, other information should be needed to maintain the self-motion perception during steady-state body movements. Visual information of counter-motion of the visual scene is considered to play an important role in maintaining self-motion perception. Physiological evidence supports this view by indicating that visual inputs (such as optical flow caused by an observer's locomotion), as well as vestibular information, activate the vestibular nuclei (e.g., Dichgans et al., 1973; Hoffmann and Distler, 1986; for reviews see Ilg, 1997; Barmack, 2003).

Many studies have revealed that various stimulus attributes and visual conditions affect the strength and the direction of vection. For example, the strength of vection increases with increasing stimulus velocity (e.g., Brandt et al., 1973; Nakamura and Shimojo, 1999) and size (e.g., Brandt et al., 1973; Berthoz et al., 1975). In addition, non-attended visual motion modulates the direction of vection (Kitazaki and Sato, 2003).

However, to our knowledge, research on the effects of stimulus color is scarce. One of the few studies is by Bonato and Bubka (2006). In their study, circular vection (rotation vection) was measured by manipulating the color of stripes attached inside a rotating drum. There were three color conditions related to the stripes: black-and-white, gray-shade, and chromatic conditions. The stripes in the chromatic condition consisted of 6 different colored stripes, that is, blue, green, red, yellow, black, and white, and the luminance was equated in all the conditions. It was found that the chromatic stripes hastened the onset and magnitude of vection. Bubka and Bonato (2010) extended this by using videotaped scenes from the first-person perspective while walking down a corridor with (chromatic condition) or without (grayscale condition) color. They found that the chromatic display hastened the onset and magnitude of vection. Although the reason why chromatic stimuli can enhance vection is not clear from the two studies, the authors in those studies interpreted their findings in terms of two separate neurological pathways: magnocellular and parvocellular (Livingstone and Hubel, 1987, 1988). It is well known that the magnocellular pathway is highly sensitive to contrast and motion while the parvocellular pathway is sensitive to color, but not motion. From this fact, the authors concluded that the parvocellular system may, in some way, be involved in vection.

Seno et al. (2010b) examined effects of stimulus color by manipulating the color of expanding optical flows and a background. In their study, the color of the dots and background were either red or green, and the dots were equiluminant. They assumed that vection would be inhibited by red, because various psychophysical studies have revealed that red has inhibiting effects on visual performance and the findings of these studies have been considered as evidence that red inhibits motion processing in the magnocellular pathway. A series of their experiments clearly support their prediction that red dots and a red background inhibit vection. In addition, their results showed no enhancement of vection by green dots, as compared with the vection induced by white dots. In one of their experiments, the inhibitory effects of red were also observed even when the perceived motion strength was equated, which is not consistent with their explanation of the role of magnocellular system activity in vection. Based on this result, Seno et al. proposed other explanations. One such explanation is that the color of dots would affect the perceived depth of the stimulus. Research has suggested that because of the ocular chromatic aberration, red stimuli appear to be nearer than green stimuli, even when both stimuli are presented with the same depth (Winn et al., 1995). It is well known that if visual stimuli differ in depth, vection is determined by the stimulus that is more distant, or appeared to be distant (e.g., Brandt et al., 1975; Ohmi et al., 1987; Ohmi and Howard, 1988; Howard and Howard, 1994; Ito and Shibata, 2005; Seno et al., 2009). Therefore, it is possible that the inhibition of vection by red dots may have reflected the perceived depth caused by the ocular chromatic aberrations.

We decided to further examine the effects of color on vection for two reasons. First, we wanted to obtain a better understanding of the effects of a single color on vection by using various colors; that is, red, yellow, green, and blue. To our knowledge, research on the effects on vection of a single color, not multiple colors, is scarce. In addition, if vection reflects the ocular chromatic aberration proposed by Seno et al. (2010b) when a single color is used, then we can expect that vection would become stronger for blue dots than for dots of other colors, because the dots appear to be more distant for shorter wavelengths due to the ocular chromatic aberration.

Second, we wanted to examine the effects of a single color on vection by using dots with various depth cues (e.g., changing size and disparity cues). According to Bonato and Bubka (2006), a visual stimulus that shares features with a natural visual environment (e.g., multiple colors and complexity) serves as a reliable visual frame of reference and results in the enhancement of visual environment stability around an observer. Consequently, observers could interpret the stimulus motion as being caused by self-motion and not by the motion of the environment. According to this hypothesis, the single-colored dots may not have been perceived as natural enough to create a reliable visual frame of reference, thus resulting in no vection enhancement (see also Nakamura et al., 2010). However, Seno et al. (2010b) used optical flows with changing velocity cues (i.e., closer dots moved faster than distant ones) but no changing size or disparity cues. Depth is considered a feature in a natural environment (Bonato and Bubka, 2006) and enhances vection (e.g., Palmisano, 1996, 2002). It is, therefore, possible that inconsistent depth information may have reduced the reliability of the visual frame of reference derived from stimulus features. As a result, the effects of a single color on vection may have been concealed.

We conducted three experiments. In all the experiments, two types of optical flows—i.e., standard modulus and test stimulus—were sequentially presented in a trial. The participants were asked to report vection to the test stimulus relative to that of the standard modulus. In Experiment 1, we measured vection to a white or single-colored test stimulus presented on a black background. In Experiment 2, to examine the potential order effects of standard modulus and test stimulus on vection, we manipulated the color of the standard modulus and measured vection to a white test stimulus. In Experiment 3, to examine the effects of luminance artifacts between the color conditions on vection, we employed a background that had higher luminance than the dots. In Experiments 1 and 3, we used two stimulus velocities—i.e., 10 and 20 km/h—in order to examine whether the present results align with the previous findings that showed increases in vection with increasing stimulus velocity (e.g., Brandt et al., 1973; Nakamura and Shimojo, 1999).

## Experiment 1

### Materials and methods

#### Participants

Sixteen participants took part in the experiment (mean age = 22.25, *SD* = 1.68; 11 men and 5 women). They had normal or corrected-to-normal vision. All the participants gave written informed consent for participating in the experiment. The experimental protocol was approved by the ethics committee of Ritsumeikan University. The tenets of the Declaration of Helsinki were followed.

#### Apparatus and stimuli

A personal computer (Apple Mac Pro Early 2009) was used to control the experiment and generate stimuli that were rear-projected onto a screen with a 3D projector (Vivitek D795WT) with a refresh rate of 120 Hz. The size of screen area was 95 × 95 cm, subtending 72.3° × 72.3°. The stimuli were viewed binocularly with a 3D shutter goggle from a viewing distance of 65 cm. The experimental program was written using MATLAB with Psychophysics Toolbox extensions (Brainard, 1997; Pelli, 1997). Participants' responses were recorded by numeric keypad at a sampling rate of 60 Hz.

**Figure 1:**
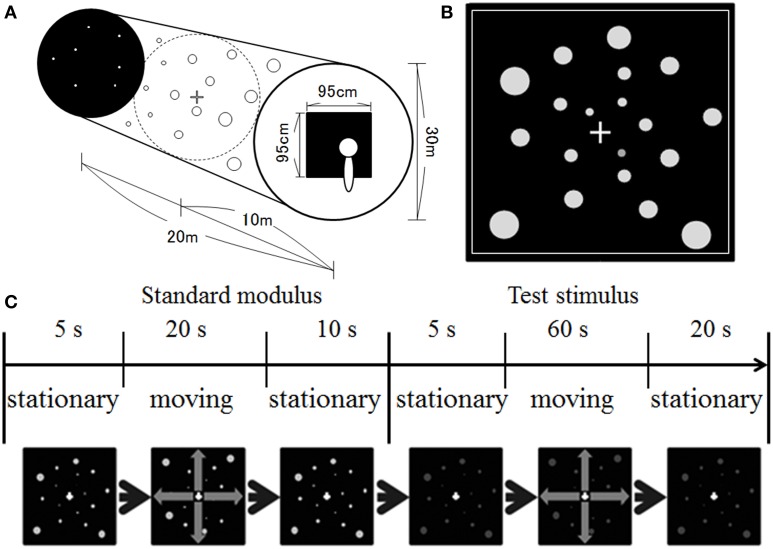
**Illustration of (A) simulated space, (B) stimulus display, and (C) time course of stimulus presentation used in the present study**.

Figure 1 illustrates our stimulus display. We simulated a space spreading in depth (Figure 1A). The size of the space was 20 m in depth with a diameter of 30 m. One thousand dots were positioned randomly in the space. The dots were presented in equiluminant white (u′ = 0.188, v′ = 0.503), red (u′ = 0.388, v′ = 0.531), yellow (u′ = 0.213, v′ = 0.551), green (u′ = 0.148, v′ = 0.540), or blue (u′ = 0.150, v′ = 0.335) on a black background. The dots' luminance measured through the goggles was around 1.21 cd/m^2^ and 0.66 cd/m^2^ on the central and 30° peripheral screens, respectively. The luminance of the background was around 0.15 cd/m^2^ and 0.05 cd/m^2^ on the central and 30° peripheral screen, respectively. The position of each dot was first selected in a way that the motion path strayed from the observer's face (dot was simulated to move at least 10 cm away from line of sight). The position of each dot was refreshed at a rate of 60 Hz, and each dot was simulated to move in depth (either approaching or receding) by manipulating uncrossed disparity defined by its distance and the distance of the screen from the observer. The maximum uncrossed disparity was 5.53°. When the dots moved in a space between the screen and the observer, a crossed disparity was added to them. Since the position of each dot was randomly selected first, most dots disappeared from the screen before moving a space between the observers and the screen. However, if a dot position was selected for that dot to move on the path 10 cm away from the line of sight, the maximum crossed disparity was 14.45° (corresponding to approximately 18.3 cm from the observer). It should be noted that, in the present study, the participants would not be capable of fusing some dots, particularly for too near dots. However, in daily life situations, there are many objects including too near or too far objects in the visual field. Therefore, the presence of unfused images in the visual field would be consistent with the visual features in daily life situations. A square frame in white surrounded the screen area in which the stimuli were presented (see Figure 1B). This frame served as a window through which the observer could see outside. The dots disappeared when they reached the edge of the space and reappeared at the opposite side. The size of the dots was manipulated to change according to a simulated distance from an observer. The simulated dot was 4 cm in diameter. The velocity of dot motion was either 10 or 20 km/h.

A 1 × 1 cm white fixation cross was presented at the center of the display. The fixation cross was presented in the middle of the space.

#### Procedure

The experiment was conducted in a dark booth. The participants were seated comfortably with their heads upright. No apparatus was used to support head position, and the participants maintained their postures and head positions by themselves during the experimental sessions. After 3 min of darkness adaptation, the experimental session began. At the beginning of each trial, stationary white dots were presented for 5 s, after which they moved at a velocity of 10 km/h for 20 s (i.e., standard modulus), followed by the presentation of stationary dots for 10 s (see Figure 1C). After the offset of the standard modulus, stationary achromatic or chromatic dots were presented for 5 s, after which they moved for 60 s (i.e., the test stimulus), followed by the presentation of stationary dots for 20 s. Note that although the standard modulus always moved at 10 km/h, the direction was the same as that for the test stimulus.

The participants were asked to press one of three buttons and to keep it pressed whenever they felt vection. The buttons corresponded to the strength of vection: strong, same, or weak relative to the vection induced by the standard modulus. After each trial, the participants were asked to report the direction of vection and to rate, with numeric values, the average strength of vection during the test stimulus presentation period relative to what was seen during the standard modulus presentation period. The participants were told to assign a value of 100 for vection as strong as that induced by the standard modulus and 0 for no vection. Because vection induced by the test stimulus was defined by that induced by the standard modulus in the present study, we also asked the participants to report whether they felt vection in response to the standard modulus after each trial. There were two trials for each combination of the five color and four velocity conditions.

To examine the potential effects on vection of standard modulus adaptation, we also conducted a control condition in which stationary dots were presented, as a test stimulus, for 60 s after the presentation of the standard modulus. Note that the standard modulus was always approaching the participants. In all the other aspects, the method was identical to that used in the experimental conditions.

There were 50 trials in total: 40 experimental and 10 control trials. The order of the color and velocity conditions (including the control condition) was randomized across the participants. There were five sessions of 10 trials each. All the participants completed their trials over 2 days, depending on their schedules and availability. There were 30-min rest periods between sessions. Before the experiment, all participants practiced the task. In the practice session, we first presented the participants with the standard modulus alone several times and asked them to report whether they felt vection. After we confirmed that the standard modulus was capable of inducing vection, the participants performed several (about 10 trials) trials to learn the task and magnitude estimation (ME) values. The color and velocity conditions during this session were randomly selected. After the rest period (of longer than 20 min), the experimental session was conducted.

#### Key press data analyses

From the key press data, we calculated vection-onset latency and vection duration. Vection-onset latency was defined as the time elapsed from the initiation of flow motion to when the participant first pressed one of three buttons. In the control condition only, no vection to the test stimulus was reported. Therefore, in this condition, as in previous studies (e.g., Palmisano et al., 2003; see also Telford et al., 1992), we assigned a value of 60 s to the latency, which was equal to the whole duration of the optical-flow stimulation. In the analysis of vection duration, total duration of vection, as well as the weak-, same-, and strong-vection durations were calculated. Total-vection duration was the duration of key press. The weak-, same-, and strong-vection durations were calculated as the duration for which each button was pressed, respectively.

### Results

In all except for the control condition, the participants consistently reported vection depending on the flow motion direction. For example, when the dots appeared to be approaching, they reported forward vection. There was no trial in which the participants felt no vection to the standard modulus.

**Figure 2:**
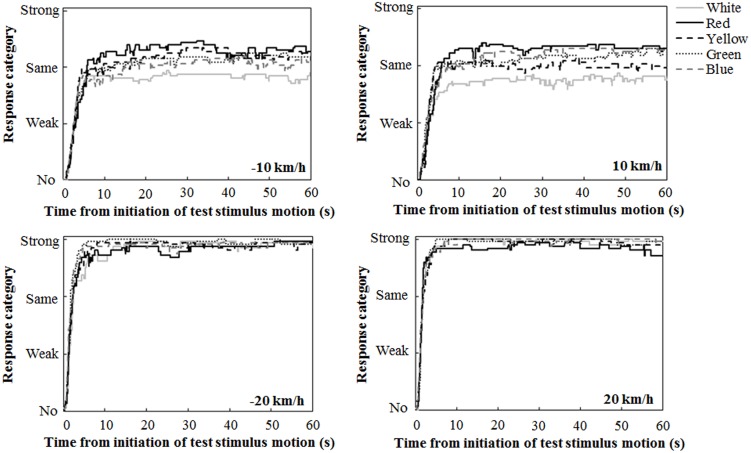
**Mean traces of key press data during the 60-s interval of the test stimulus presentation**.

Figure 2 illustrates the mean traces of key press data during the 60-s interval of the test stimulus presentation, calculated by assigning values of 0, 1, 2, and 3 for response categories of no, weak, same, and strong vections, respectively. A close inspection revealed the tendency that at ±10 km/h, the participants reported strong vection more frequently in the chromatic conditions than in the achromatic condition. The participants also tended to report strong vection more frequently in the red condition than in the other chromatic conditions.

#### Vection duration

**Figure 3:**
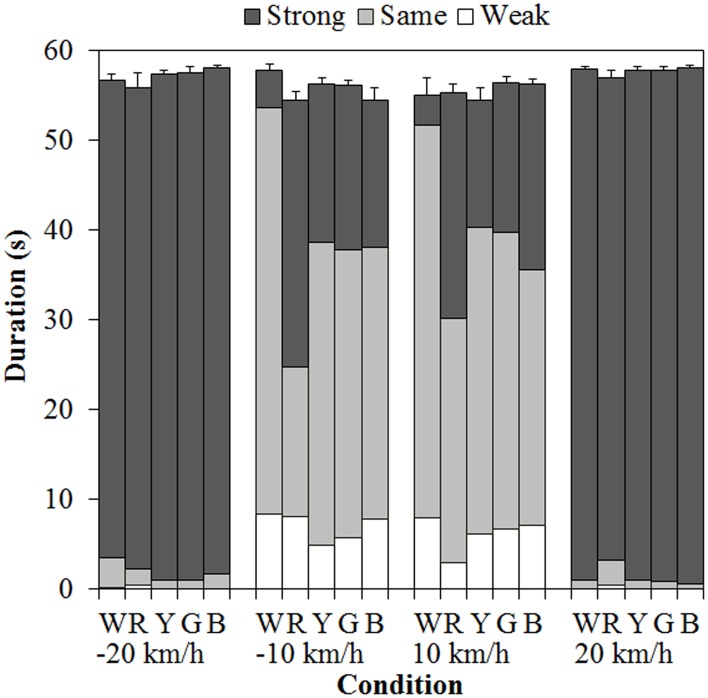
**Mean vection duration in Experiment 1.** W, R, Y, G, and B on the horizontal axis indicate white, red, yellow, green, and blue, respectively. Error bars indicate SE of the total durations.

Figure 3 shows the results of mean vection duration. Each component of the bar chart indicates the mean weak-, same-, and strong-vection durations, respectively. Positive values on the horizontal axis mean that the background dots were approaching, and negative values mean that the background dots were receding. As shown in the figure, total-vection durations of the three components and weak-vection durations did not differ by the stimulus color. On the other hand, same-vection durations and strong-vection durations were affected by the color of the dots, particularly at ±10 km/h.

A 5 (color) × 4 (velocity) ANOVA for each duration measure revealed significant main effects of color in the same- [*F*_(4, 60)_ = 5.740, η^2^_*p*_ = 0.277, *p* = 0.0006] and strong-vection durations [*F*_(4, 60)_ = 4.392, η^2^_*p*_ = 0.226, *p* = 0.0035] but did not in the total- and weak-vection durations. The main effects of velocity were significant in all the duration measures [total-vection duration, *F*_(3, 45)_ = 10.641, η^2^_*p*_ = 0.415, *p* < 0.0001; weak-vection duration, *F*_(3, 45)_ = 9.702, η^2^_*p*_ = 0.393, *p* < 0.0001; same-vection duration, *F*_(3, 45)_ = 82.367, η^2^_*p*_ = 0.846, *p* < 0.0001; and strong-vection duration, *F*_(3, 45)_ = 201.019, η^2^_*p*_ = 0.931, *p* < 0.0001]. In addition, the interactions between color and velocity were significant in the same- [*F*_(12, 180)_ = 3.215, η^2^_*p*_ = 0.177, *p* = 0.0003] and strong-vection durations [*F*_(12, 180)_ = 4.048, η^2^_*p*_ = 0.213, *p* < 0.0001].

*Post-hoc* analyses by the Ryan's method (Ryan, 1960) showed that the same-vection durations for the achromatic (white) dots were significantly longer than those for the chromatic dots (all *p*s < 0.05). The strong-vection durations were significantly longer for the chromatic dots than for the achromatic dots (all *p*s < 0.05). *Post-hoc* analyses for the effect of velocity showed significant differences between low and high velocities (all *p*s < 0.05) in all the measures.

Subsequent analyses of the interaction showed significant simple main effects of color at −10 km/h and 10 km/h in both the same- [−10 km/h, *F*_(4, 240)_ = 11.240, η^2^_*p*_ = 0.158, *p* < 0.0001; 10 km/h, *F*_(4, 240)_ = 4.626, η^2^_*p*_ = 0.072, *p* = 0.0013] and strong-vection durations [−10 km/h, *F*_(4, 240)_ = 8.884, η^2^_*p*_ = 0.129, *p* < 0.0001; 10 km/h, *F*_(4, 240)_ = 7.318, η^2^_*p*_ = 0.109, *p* < 0.0001]. In the same-vection durations at −10 km/h, the duration was the shortest for the red dots (all *p*s < 0.05) and it was shorter for the other chromatic dots than for the achromatic dots (all *p*s < 0.05). At 10 km/h, the duration was significantly shorter for the red and blue dots than for the white dots (both *p*s < 0.05). In the strong-vection durations at −10 km/h, the duration was the longest for the red dots (all *p*s < 0.05) and it was also longer for the other colored dots than for the white dots (all *p*s < 0.05). At 10 km/h, the strong-vection duration was longer for the colored dots than for the white dots (all *p*s < 0.05).

#### Vection-onset latency

**Figure 4:**
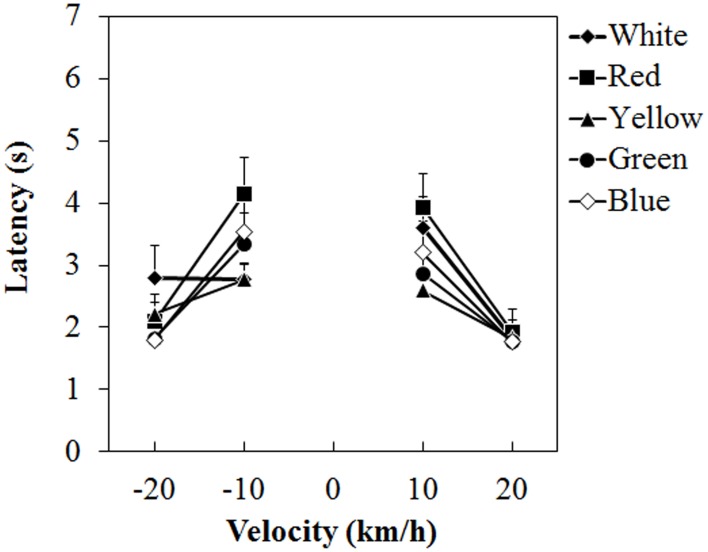
**Mean latency in Experiment 1.** Error bars indicate SE.

Figure 4 shows the mean vection-onset latency. A Two-Way ANOVA showed no main effect of color. The effect of velocity was significant [*F*_(3, 45)_ = 35.212, η^2^_*p*_ = 0.701, *p* < 0.0001]. There was a significant interaction between color and velocity [*F*_(12, 180)_ = 2.193, η^2^_*p*_ = 0.127, *p* = 0.0138]. *Post-hoc* analyses showed significant differences between low and high velocities (all *p*s < 0.05). Subsequent analyses of the interaction showed a significant simple main effect of color at -10 km/h [*F*_(4, 240)_ = 3.826, η^2^_*p*_ = 0.060, *p* = 0.0049] and 10 km/h [*F*_(4, 240)_ = 3.299, η^2^_*p*_ = 0.052, *p* = 0.0118]. At −10 km/h, the latency was significantly longer for the red dots than for the white and yellow dots (both *p*s < 0.05). At 10 km/h, the latency was longer for the red dots than for the yellow dots (*p* < 0.05).

#### Magnitude estimation

**Figure 5:**
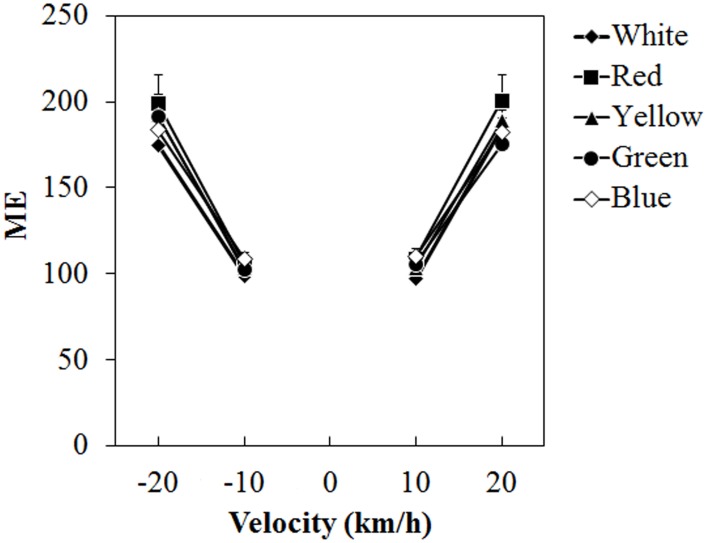
**Mean magnitude estimation (ME) in Experiment 1.** Error bars indicate SE.

Figure 5 shows the mean ME. A Two-Way ANOVA showed no main effect of color. The effect of velocity was significant [*F*_(3, 45)_ = 65.076, η^2^_*p*_ = 0.813, *p* < 0.0001]. There was a significant interaction between color and velocity [*F*_(12, 180)_ = 1.861, η^2^_*p*_ = 0.110, *p* = 0.0419]. *Post-hoc* analyses showed significant differences between low and high velocities (all *p*s < 0.05). Subsequent analyses of the interaction showed a significant simple main effect of color at −20 km/h [*F*_(4, 240)_ = 2.960, η^2^_*p*_ = 0.047, *p* = 0.0205] and 20 km/h [*F*_(4, 240)_ = 2.924, η^2^_*p*_ = 0.046, *p* = 0.0218]. At −20 km/h, ME was significantly larger for the red dots than for the white dots (*p* < 0.05) and it was significantly larger for the red dots than for the green dots at 20 km/h (*p* < 0.05).

#### Control condition

In the control trials, mean total-, weak-, same-, and strong-vection durations were 0 s in all the color conditions. According to the definition in previous studies, therefore, mean latency was 60 s. The mean ME was 0 in all the conditions except for the green dots (mean = 0.63), as one participant reported very slight vection.

### Discussion

The duration results showed that chromatic stimuli hastened vection magnitude (indicated by longer strong-vection durations and shorter same-vection durations) as compared to those induced by achromatic stimuli. At low velocities (10 km/h and −10 km/h), red dots induced stronger vection than did the other chromatic dots, which is not consistent with the findings of Seno et al. (2010b) or the prediction made based on the ocular chromatic aberrations. The ME results showed a tendency of larger vection magnitude for the red dots than for the white dots at high velocity condition (i.e., 20 and −20 km/h). These results are similar to the findings of Bonato and Bubka (2006) and Bubka and Bonato (2010). As we discussed earlier, these two studies used stimuli with multiple colors, not a single color. Seno et al. (2010b) used single-colored stimuli and reported inhibition and no enhancement of vection by red and green, respectively. Therefore, the present study is the first to report vection enhancement by single-colored stimuli. This finding suggests that the addition of a single color in the visual field can enhance vection, at least when various depth cues are available.

The reason why single stimulus color enhanced vection in our study is not clear. One possibility is the reliability of visual frame of reference. As we mentioned in the Introduction, a visual stimulus that shares natural features forms a reliable frame of reference (e.g., Bonato and Bubka, 2006). Since the present study used various depth cues (cf. Seno et al., 2010b), consistent depth information may have enhanced the reliability of visual frame of reference based on stimulus features. As a result, the participants' interpretation of the environment stability may have been susceptible to stimulus features. Although single-colored stimuli are less common in nature than multiple colored stimuli are, they may have been perceived as being more natural than achromatic stimuli, resulting in stronger vection.

Another possibility is the dot visibility. In the present study, we presented dots of one of five colors on a black background, and the color difference from the background color varied between the color conditions, although the luminance of the dots and the luminance's contrast with the background were equated as much as possible between the conditions. Table 1 shows the Euclidean distance between the dot colors and the background color on a uniform chromaticity scale diagram (u′, v′). As shown in the table, the distance was the longest for the red dots, which is consistent with the strong-vection duration results. If we assume that the larger color difference caused higher dot visibility, this may account for the present results. It should be noted that although the dots' visibility due to the color difference with the background may account for the present findings, it does not account for the findings of Seno et al. (2010b) showing no vection enhancement by single-colored dots irrespective of the background color. The possibility of dot visibility is further discussed in the General Discussion together with the results of Experiment 3.

**Table 1:** **Euclidean distance between the dot colors and the background color on a uniform chromaticity scale diagram (u′, v′)**.

**Color of dots**	**ΔE_u_′v′**
White	0.0152
Red	0.2169
Yellow	0.0620
Green	0.0435
Blue	0.1601

The present results further showed longer latencies for the red dots than for the other dots, which is consistent with Seno et al. (2010b). This cannot be explained by the ideas mentioned above. One possibility is that the latency reflected the sensitivity of the light-sensitive rods on the retina, not the color-sensitive cones. It is well known that the stimulation of the peripheral retina is important to induce vection (e.g., Brandt et al., 1973; Berthoz et al., 1975; see also, Andersen and Braunstein, 1985). In general, rods are concentrated on the peripheral retina and more sensitive to shorter wavelengths (see Sagawa and Takeichi, 1987; Anstis, 2002). On the other hand, cone density rapidly decreases with increasing distance from the fovea, and cones are more sensitive to longer wavelengths. Under the light-adapted condition, rods are less sensitive than cones. As luminance decreases, rods gradually become sensitive. As a result, the eye becomes less sensitive to longer wavelengths than to shorter wavelengths (Purkinje phenomenon, Purkinje, 1825; see also Sagawa and Takeichi, 1987; Anstis, 2002). In the present study, the luminance of the dots and the background used was quite low (see the Materials and Methods section), although the stimulus color was perceptible. Therefore, the participants were considered to be in mesopic vision, and rod signals (as well as cone signals) would affect detection and/or motion perception of dots. As a result, the participants were less sensitive to the red dots, resulting in longer latency of vection onset. It should be noted that the rod contribution accounts for the result of latency, but not for the duration and ME results, because duration measures and ME suggest stronger vection for the red dots than for the other dots.

Although the modulations of vection by dot color were manifest at the low velocities (±10 km/h), they were not at the higher velocities (±20 km/h). This discrepancy could be due to ceiling and floor effects for duration measures used in the present study. In the present study, stimuli with depth cues were used and moved at relatively high velocities (10 and 20 km/h). It has been demonstrated that strength of vection increased with increasing depth perception (Andersen and Braunstein, 1985; Palmisano, 1996, 2002) and velocity (Nakamura and Shimojo, 1999). Therefore, vection indicated by latency and durations would have been saturated at those velocities. It should be noted that the ME at ±20 km/h tended to be larger with chromatic dots than with achromatic dots, while it was not different between color conditions at ±10 km/h.

In this experiment, the white dots (i.e., the standard modulus) were always presented before the white or single-colored test stimulus. Therefore, the weak vection to the white dot motion may have reflected the adaptation to the standard modulus motion. If this were true, the vection to the stationary white test stimuli would occur in the opposite direction to the standard modulus motion. The results of the control condition showed no vection, irrespective of the test stimulus colors. In addition, in the present study, stationary dots were presented for 15 s before the presentation of the test stimulus motion (see the Materials and Methods section). Therefore, it is unlikely that adaptation to the standard modulus motion could account for the present results. However, it is still possible that the potential order effects of stimulus presentation influenced the present results. In Experiment 2, we examined this possibility by presenting a white or single-colored standard modulus. In this experiment, white dots were used as a test stimulus. If a single-colored stimulus can induce stronger vection than an achromatic stimulus does, then vection during the presentation of the white test stimulus should be weak relative to that during the presentation of the chromatic standard modulus, resulting in longer weak-vection durations and lower MEs for the chromatic conditions than for the achromatic condition of the standard modulus.

## Experiment 2

### Materials and methods

This experiment's method was the same as Experiment 1's with the following exceptions. First, the color of the standard modulus was white, red, yellow, green, or blue. Second, the color of the test stimulus was always white, and the stimulus moved at ±10 km/h. Finally, we did not conduct a control condition in which stationary dots were presented during the test stimulus presentation period.

Twelve participants took part in this experiment (mean age = 23.08, *SD* = 1.38; 9 men and 3 women), 10 of whom participated in Experiment 1. There were 20 trials in all, with two trials for each combination of the 5 color and 2 velocity conditions.

### Results and discussion

**Figure 6:**
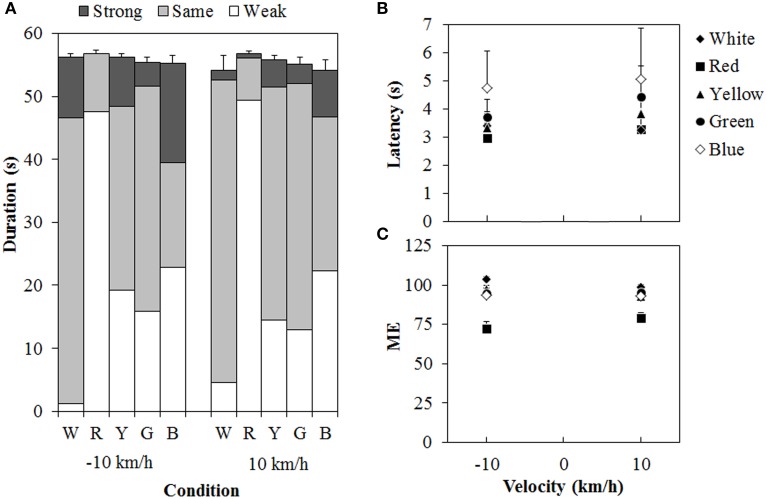
**Results of (A) mean vection duration, (B) mean latency, and (C) mean magnitude estimation (ME) in Experiment 2.** W, R, Y, G, and B on the horizontal axis in **(A)** indicate white, red, yellow, green, and blue condition of the standard modulus, respectively. Error bars indicate SE of the total duration. In **(B,C)**, error bars indicate SE.

Figure 6 shows the results of Experiment 2. A 5 (color of standard modulus) × 2 (velocity) ANOVA for each duration measure (Figure 6A) revealed significant main effects of color in the weak- [*F*_(4, 44)_ = 26.162, η^2^_*p*_ = 0.704, *p* < 0.0001], same- [*F*_(4, 44)_ = 19.419, η^2^_*p*_ = 0.638, *p* < 0.0001], and strong-vection durations [*F*_(4, 44)_ = 2.590, η^2^_*p*_ = 0.191, *p* < 0.0495], but not in the total-vection durations. The main effect of velocity was significant only for the strong-vection durations [*F*_(1, 11)_ = 5.635, η^2^_*p*_ = 0.339, *p* = 0.0369], showing stronger vection at −10 km/h than at 10 km/h. This result is consistent with the previous findings (e.g., Ito and Shibata, 2005; Seno et al., 2010a). There was no significant interaction for any of the duration measures. *Post-hoc* analyses showed that weak-vection durations were longer in the chromatic conditions than in the achromatic condition (all *p*s < 0.05). They were also longer in the red condition than in the other chromatic conditions of the standard modulus (all *p*s < 0.05). The same-vection durations showed a pattern opposite to that of the weak-vection durations. They were significantly shorter in the chromatic conditions, except for the green condition, than in the achromatic condition (all *p*s < 0.05). They were also significantly shorter in the red condition than in the yellow, green, and blue conditions (all *p*s < 0.05). The durations were significantly shorter in the blue condition than in the green condition (*p* < 0.05). The strong-vection durations showed significantly longer durations in the blue condition than in the red condition. The results of duration measures clearly suggest that the vection to the single-colored standard modulus was stronger than that to the white test stimulus, and that the red dots induced stronger vection than the other-colored dots did, thus supporting Experiment 1's findings.

For the latency (Figure 6B), a Two-Way ANOVA revealed no main effect or interaction between the variables, and this suggested no difference in the vection onset latency to the white test stimulus between the standard modulus conditions. For the ME (Figure 6C), a Two-Way ANOVA revealed only a significant main effect of color [*F*_(4,44)_ = 22.595, η^2^_*p*_ = 0.673, *p* < 0.0001]. The interaction between the variables was not significant. *Post-hoc* analyses showed significantly lower MEs in the red condition than in the other conditions (all *p*s < 0.05). This suggests, again, that the vection was stronger for the red dots than for either the white or the other-colored dots.

## Experiment 3

In Experiments 1 and 2, the dot luminance was equated between the color conditions as much as possible. However, because of the limitations of the apparatus used, it is still possible that the dot luminance was higher in the chromatic conditions than in the achromatic condition, thus resulting in higher contrast between the dots and the background. If so, it can be expected that vection to the chromatic dots became weaker than that to the achromatic dots when the dots were presented on a background with higher luminance than the dot luminance, because the luminance artifact should produce lower luminance contrasts between the dots and the background in the chromatic conditions than in the achromatic condition. In Experiment 3, we investigated this possibility.

### Materials and methods

The method used was the same as that of Experiment 1, except for the following changes. First, the luminance of the background measured through the goggles was around 2.21 cd/m^2^ and 1.42 cd/m^2^ on the central and 30° peripheral screens, respectively. Note that in this experiment, white dots appeared gray (not white) since the dot luminance of each color condition was identical to that used in Experiment 1. We selected background luminance that was slightly higher than that of the dots, because the luminance difference between the color conditions, if any, would sufficiently produce the differences in the contrasts between the dots and the background.

Second, after each trial, the participants were asked to rate both the visibility and the perceived velocity of the dot motion, because the use of a bright background could make the visibility and perceived velocity of dot motion lower for the chromatic than for the achromatic dots. The participants were told that the visibility and perceived velocity to the standard modulus were set to 100. In this experiment, we did not measure ME of vection strength in order to avoid a possible confusion (although we asked the participants to report the direction of vection). Finally, we did not conduct a control condition in which stationary dots were presented during the test stimulus presentation period.

Twelve participants who participated in Experiment 2 took part in this experiment. There were 40 trials in all, with two trials for each combination of the 5 color and 4 velocity conditions.

### Results

**Figure 7:**
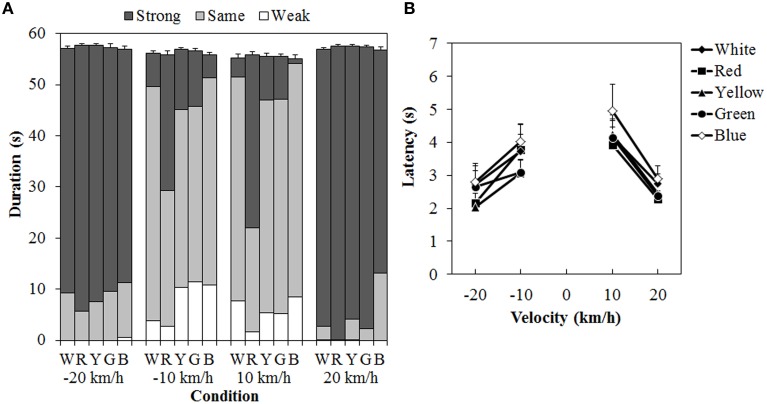
**Results of (A) mean vection duration and (B) mean latency in Experiment 3.** W, R, Y, G, and B on the horizontal axis in **(A)** indicate white (gray), red, yellow, green, and blue, respectively. Error bars indicate SE of the total duration. In **(B)** error bars indicate SE.

One participant was removed from a subsequent analysis because that person could not perceive vection at all to either the standard modulus or the test stimulus. Figure 7 shows the results of the duration measures and latency. A 5 (color) × 4 (velocity) ANOVA for each duration measure (Figure 7A) showed significant main effects of color in the total- [*F*_(4, 40)_ = 3.129, η^2^_*p*_ = 0.238, *p* = 0.0249], same- [*F*_(4, 40)_ = 11.446, η^2^_*p*_ = 0.534, *p* < 0.0001], and strong-vection durations [*F*_(4,40)_ = 13.134, η^2^_*p*_ = 0.568, *p* < 0.0001], but not in the weak-vection duration. The main effect of velocity was significant for all duration measures [total-vection duration, *F*_(3, 30)_ = 10.156, η^2^_*p*_ = 0.504, *p* = 0.0001; weak-vection duration, *F*_(3, 30)_ = 4.232, η^2^_*p*_ = 0.297, *p* = 0.0131, same-vection duration, *F*_(3, 30)_ = 29.666, η^2^_*p*_ = 0.748, *p* < 0.0001; strong-vection duration, *F*_(3, 30)_ = 55.113, η^2^_*p*_ = 0.846, *p* < 0.0001]. The interaction between color and velocity was significant only in the strong-vection duration [*F*_(12,120)_ = 3.273, η^2^_*p*_ = 0.247, *p* = 0.0004].

*Post-hoc* analyses showed that the total-vection durations were significantly shorter for the blue dots than for the yellow dots (*p* < 0.05). The same-vection durations were significantly shorter for the red dots than for the other dots (all *p*s < 0.05). The strong-vection durations were significantly longer for the red dots than for all the other dots (all *p*s < 0.05). *Post-hoc* analyses for velocity effect showed significant differences between low and high velocities (all *p*s < 0.05) in the total-, same-, and strong-vection durations. In the weak-vection duration, the durations were significantly longer at −10 km/h than at 20 and −20 km/h (both *p*s < 0.05).

Subsequent analyses for the interaction in the strong-vection duration showed significant main effects of color at velocities of −10 km/h, *F*_(4, 160)_ = 7.185, η^2^_*p*_ = 0.152, *p* < 0.0001, 10 km/h, *F*_(4, 160)_ = 16.651, η^2^_*p*_ = 0.294, *p* < 0.0001, and 20 km/h, *F*_(4, 160)_ = 2.647, η^2^_*p*_ = 0.062, *p* = 0.0354. At the velocities of −10 km/h and 10 km/h, the strong-vection duration was significantly longer for the red dots than for the other dots (all *p*s < 0.05). At the velocity of 20 km/h, the duration was significantly longer for the red dots than for the blue dots (*p* < 0.05).

For the latency (Figure 7B), a Two-Way ANOVA showed significant main effects of color, *F*_(4, 40)_ = 3.914, η^2^_*p*_ = 0.281, *p* = 0.0090, and velocity, *F*_(3, 30)_ = 9.243, η^2^_*p*_ = 0.480, *p* = 0.0002. There was no significant interaction between the two. *Post-hoc* analyses for the effect of color showed significantly longer latency for the blue dots than for the red, yellow, and green dots (all *p*s < 0.05). *Post-hoc* analyses for the effect of velocity showed significantly longer latencies at 10 km/h than at −20 and 20 km/h (both *p*s < 0.05).

**Figure 8:**
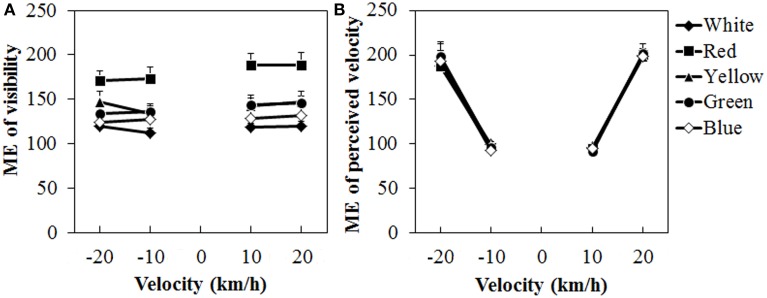
**Results of mean magnitude estimation (ME) of (A) visibility and (B) perceived velocity in Experiment 3.** Error bars indicate SE.

Figure 8 shows the mean visibility and perceived velocity of dot motion. As seen in the figure, visibility was higher for the red dots than for the other dots, while perceived velocity did not change much with differences in dot colors. A Two-Way ANOVA for visibility (Figure 8A) showed only a significant main effect of color, [*F*_(4, 40)_ = 15.691, η^2^_*p*_ = 0.610, *p* < 0.0001]. There was no interaction between the two. *Post-hoc* analyses showed that visibility was higher for the red dots than for the other dots (all *ps < 0.05*). The visibility was also significantly higher for the yellow dots than for the white dots (*p* < 0.05).

For the perceived velocity (Figure 8B), a Two-Way ANOVA showed no main effect of color. The effect of velocity was significant, [*F*_(3, 30)_ = 76.180, η^2^_*p*_ = 0.884, *p* < 0.0001]. There was no interaction between the two variables. *Post-hoc* analyses showed significant differences between low and high velocities (all *p*s < 0.05).

### Discussion

According to the prediction derived from the luminance artifact, vection should be lower for the chromatic than for the achromatic dots. The results of the present experiment showed that at low velocity conditions (±10 km/h), the red dots still increased the vection magnitude (indicated by longer strong-vection durations and shorter same-vection durations) as compared to those induced by the achromatic or other chromatic dots. The differences between the achromatic dots and the other chromatic dots were not significant. However, on average, the strong-vection durations were longer, and the same-vection durations were shorter, for the yellow and green dots than for the white (gray) dots. Taken together, these results suggest that the luminance artifact cannot account for the enhancement of vection by single-colored dots in Experiment 1.

The visibility results showed that the red dots were more visible than either the achromatic or the other chromatic dots. This did not support the prediction concerning the luminance artifact. Again, it is unlikely that the luminance artifact would account for the results of Experiment 1. The dot luminance was equated as much as possible while the color difference varied between the conditions (see Table 1). Therefore, the higher visibility for the red dots than for the other dots could be partially explained by the color difference.

The results also showed longer latencies for the blue dots than for the other chromatic dots at ±10 km/h. This is inconsistent with the result of Experiment 1. However, this could be explained by rod sensitivity. In the present experiment, we used a brighter background than that used in Experiment 1. Considering Purkinje phenomenon, the beneficial effects of rod sensitivity under the low luminance situation may have been eliminated for the blue dots, resulting in the long latency. This possibility also explains the relatively short strong-vection durations and low visibility for the blue dots, as compared with those for the other chromatic dots.

## General discussion

In the present study, we examined the effects of single color on vection by using optical flow with depth cues—that is, motion velocity cues, changing size cues, and changing disparity cues. The results of the duration measures (i.e., strong-, same-, and weak-vection durations) in the three experiments showed that the single-colored dots could enhance vection, at least when various depth cues were available. Experiment 1 showed longer strong-vection durations for the chromatic dots than for the achromatic dots. In Experiment 2 showed longer weak-vection durations for the white test stimulus in the chromatic conditions than in the achromatic condition of the standard modulus. Although Experiment 3 showed only a significant difference between the white (gray) and red dots, the strong-vection durations, on average, were longer for the yellow and green dots than for the white (gray) dots. In addition, the results of the three experiments showed that the red dots induced stronger vection than did the other chromatic dots, which is inconsistent with the prediction concerning the ocular chromatic aberration. Taken together, the enhancement of vection by stimulus color observed in the present study cannot be explained by the order effects of stimulus presentation and/or the luminance artifact between the dot color conditions.

Longer latency was found for the red dots than for the other dots in Experiment 1 and for the blue dots in Experiment 3. The assumption that rods would become less sensitive with increasing stimulus luminance can account for these results. As we discussed in Experiment 1, the rod contribution does not account for the whole pattern of the present results. For example, according to this explanation, the strong-vection duration should be longer for the green and blue dots than for the red dots. Our results showed longer strong-vection durations for the red dots than for dots of other colors. It is thus likely that the vection observed in the present study should reflect the relatively complex effects produced by several factors such as cognitive factors (i.e., the reference frame of the stable environments), dot visibility, and rod sensitivity under mesopic vision. We speculate that the effects of stimulus color (and color differences between the dots and background) observed in the duration measures and ME may be related to the parvocellular pathway. On the other hand, motion perception and/or detection of dots derived from rod signals may have activated the magnocellular pathway, and this may have resulted in the variations in vection onset latency. Further studies will be needed to explore this point.

In Experiment 3, dot visibility was the highest and vection the strongest for the red dots, suggesting the dependency of vection on dot visibility. As we discussed in Experiments 1 and 3, the color difference between the dots and the background may have affected the dot visibility, although this cannot account for the findings of Seno et al. (2010b). To further examine the effects of dot visibility on vection, we conducted an additional experiment in which six participants (who had participated in Experiment 3) were presented with stimuli identical to those used in Experiment 1, except that the dots moved at ±10 km/h. Vection (i.e., durations and latency) and visibility were measured. The duration and latency results were similar to those in Experiment 1 (see Supplementary Figures 1A,B). The duration results suggest the strongest vection for the red dots. However, visibility was highest for the blue dots (Supplementary Figure 1C). These results suggest that dot visibility alone cannot account for the pattern of the present results.

Two additional points concerning the present study are noteworthy. The first is that the present study showed inconsistencies between the vection measures used. In Experiment 1, for example, we found clear effects of single color in latency and duration measures at ±10 km/h with no differences between the color conditions at ±20 km/h while ME showed differences only at ±20 km/h. As we discussed in Experiment 1, latency and duration measures may have been subject to the ceiling and floor effects, resulting in a lack of difference between the color conditions at ±20 km/h. On the other hand, in the present study, the participants were asked to determine ME values after each trial by taking average vection strength over the test stimulus period; they were able to report vection by changing the key of vection strength category from time to time. The ME may not have been sensitive to detecting the effects of single stimulus color on vection observed in this study. This possibility may partially explain the discrepancy in the results of ME between Experiments 1 and 2. No significant difference was found between the stimulus color at ±10 km/h in Experiment 1 while ME differed by dot color in Experiment 2. It should be noted that several studies have reported discrepancies between the measures used (e.g., latency and ME, see Brandt et al., 1973), implying that each vection measure reflects somewhat different aspects of vection. Second, the present results did not support the findings of Seno et al. (2010b), who showed inhibition and no enhancement of vection by red and green, respectively. At this point, we do not have a conclusive explanation. However, there were several methodological differences—namely, the depth cues and the stimulus luminance used. In future studies, the discrepancies between the present results and those of Seno et al. will need to be examined.

In conclusion, the present study demonstrated that, when various depth cues are available, vection can be enhanced by the addition of single color to optical flow, which is similar to the findings of previous studies using stimuli with multiple colors (Bonato and Bubka, 2006; Bubka and Bonato, 2010). The present results further suggest that under mesopic vision, sensitivity of rods on the peripheral retina can modulate the latency of vection onset.

### Conflict of interest statement

The authors declare that the research was conducted in the absence of any commercial or financial relationships that could be construed as a potential conflict of interest.

## Supplementary material

Supplementary Figure 1 **Results of an additional experiment in which 6 participants (5 men and 1 woman) viewed either achromatic or chromatic dots presented on a black background**. **(A)** mean vection duration, **(B)** mean latency, and **(C)** mean magnitude estimation (ME) of visibility. W, R, Y, G, and B on the horizontal axis in **(A)** indicate white, red, yellow, green, and blue, respectively. Error bars indicate SE of the total duration. In **(B,C)**, error bars indicate SE.
