# Supplementary figures and images for: Single stimulus color can modulate vection
Source: Front Psychol. 2015 Apr 10;6:406. doi: 10.3389/fpsyg.2015.00406 (PMC4392300; doi:10.3389/fpsyg.2015.00406)

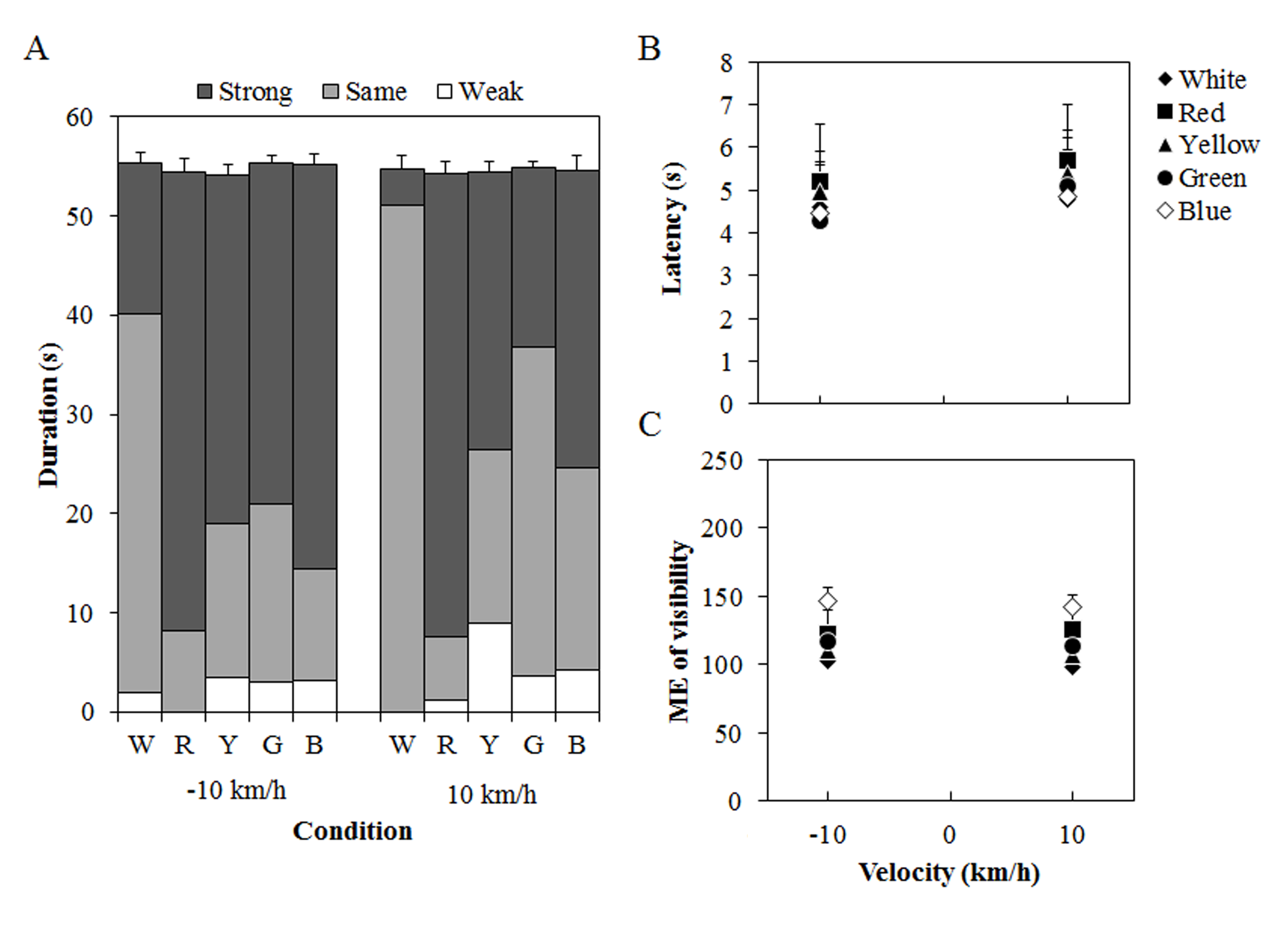

Supplement: Supplementary Figure 1 — Results of an additional experiment in which 6 participants (5 men and 1 woman) viewed either achromatic or chromatic dots presented on a black background. (A) mean vection duration, (B) mean latency, and (C) mean magnitude estimation (ME) of visibility. W, R, Y, G, and B on the horizontal axis in (A) indicate white, red, yellow, green, and blue, respectively. Error bars indicate SE of the total duration. In (B,C), error bars indicate SE. [file Image1.TIF]
